# The effect of istradefylline for Parkinson’s disease: A meta-analysis

**DOI:** 10.1038/s41598-017-18339-1

**Published:** 2017-12-21

**Authors:** Wataru Sako, Nagahisa Murakami, Keisuke Motohama, Yuishin Izumi, Ryuji Kaji

**Affiliations:** 1 Department of Clinical Neuroscience, Institute of Biomedical Sciences, Tokushima University Graduate School, Tokushima, Japan; 2 Student Laboratory, Faculty of Medicine, Tokushima University, Tokushima, Japan

## Abstract

Adenosine A_2A_ receptor antagonists are an alternative treatment strategy for Parkinson’s disease. Several randomized placebo controlled studies have tested the effect of A_2A_ receptor antagonist istradefylline, and more robust evidence has been acquired. This meta-analysis aimed to provide evidence for its efficacy and safety on patients with Parkinson’s disease. After a systematic literature search, we calculated the pooled standardized mean difference and risk ratio for continuous and dichotomous variables, respectively. Further, sensitivity analyses were performed to confirm the effect estimated by meta-analyses. Publication bias was assessed by funnel plot and deviation of intercept. Six studies satisfied our inclusion criteria. Istradefylline (40 mg/day) decreased off time and improved motor symptoms of Parkinson’s disease in homogeneous studies. Istradefylline at 20 mg/day decreased off time and improved motor symptoms, but heterogeneity was found in the analysis of the former among studies. There was a significant effect of istradefylline on dyskinesia in homogeneous studies. Publication bias, however, was observed in the comparison of dyskinesia. Other adverse events showed no significant difference. The present meta-analysis suggests that istradefylline at 40 mg/day could alleviate off time and motor symptoms derived from Parkinson’s disease. Dyskinesia might be worsened, but publication bias prevents this from being clear.

## Introduction

Parkinson’s disease (PD) is characterized by degeneration of dopaminergic neurons in the substantia nigra pars compacta (SNc), which induces motor symptoms including tremor, rigidity, akinesia, bradykinesia, and postural instability. A reduced concentration of dopamine in the striatum induces hyperactivation of the globus pallidus internus via inhibition of the direct pathway and excitation of the indirect pathway. The motor output from the striatum is considered to consist of direct and indirect pathways^1^, which mainly express dopamine D1 and D2 receptors, respectively. Recent transgenic mouse models have allowed for confirmation of the existence of two distinct pathways^2,3^.

Patients with PD are usually treated with dopamine-related drugs including levodopa, monoamine oxidase B inhibitors and dopamine agonists, which in turn increase the risk of motor and non-motor complications^4–7^. Non-dopaminergic agents are thus needed for improving PD therapy and limiting side effects. Caffeine, a non-specific adenosine A_2A_ receptor antagonist, could reduce the risk of the onset of PD and subsequent dyskinesia caused by long-term dopaminergic drug therapy^8–10^. In this context, the A_2A_ receptor antagonist istradefylline was originally developed to address motor and non-motor complications related to advanced use of dopaminergic drugs.

The effect of istradefylline was tested in several randomized placebo-controlled studies^11–17^, and was validated by other meta-analyses^18,19^. However, previous meta-analyses calculated a summary effect using the mean difference without standardization, although different estimators and subjects were involved in each study. In addition, an assessment of tolerability and publication bias and sensitivity analyses, were not performed. Furthermore, the first published meta-analysis estimated a summary effect using only three studies for each dosage, and excluded the work of Stacy *et al*.^17^ in the analysis of the effect of istradefylline (20 mg/day) on off time^18^. The second published meta-analysis combined all studies regardless of dosage, and did not assess adverse events^19^. To more robustly analyze the evidence for use of istradefylline, a detailed and systematic meta-analysis was performed.

## Methods

The general methodology is comparable to our previously published meta-analyses^20,21^.

### Study Selection

**Figure 1:**
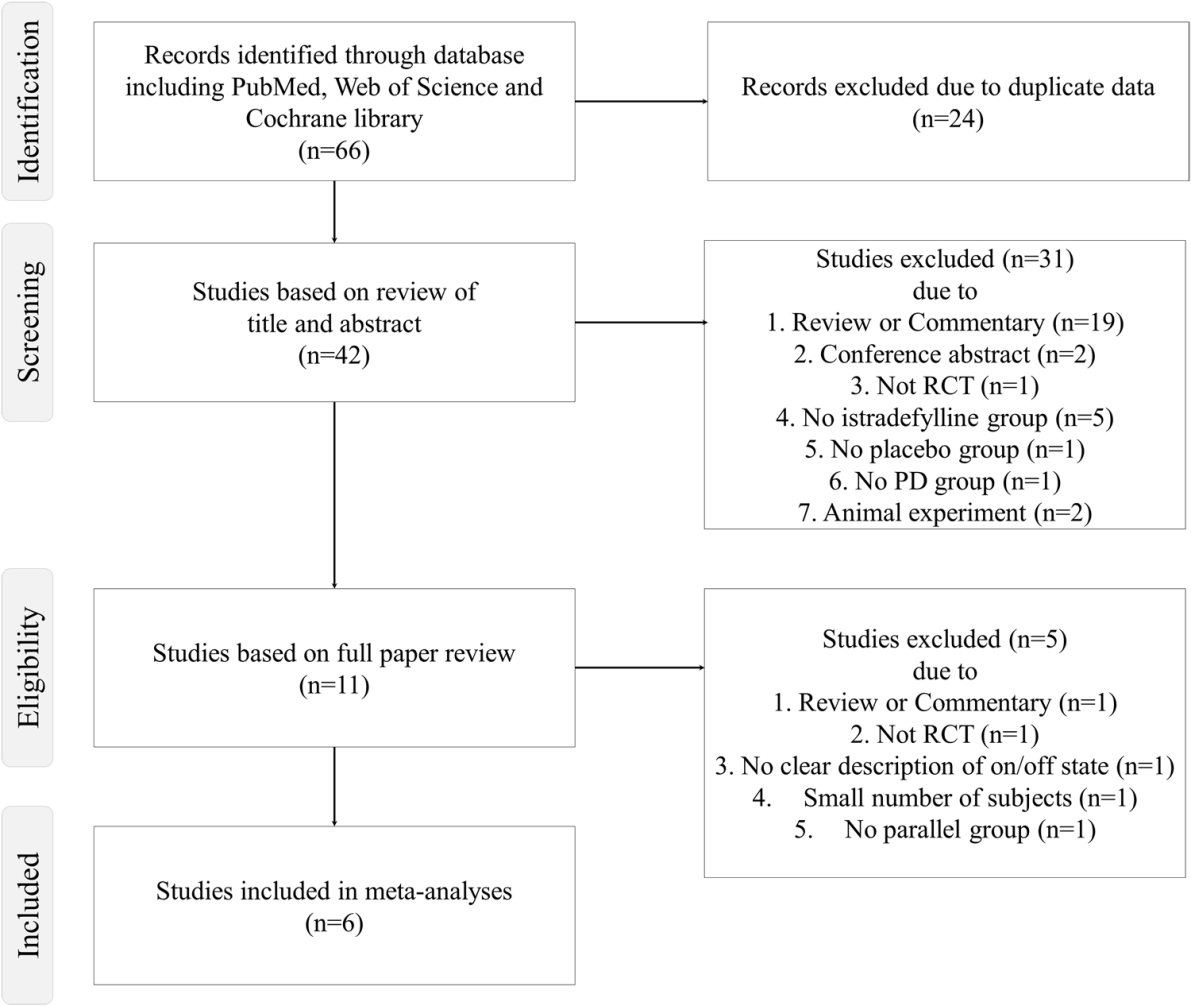
Flow chart of the inclusion process for the present meta-analysis.

Inclusion criteria in the present meta-analysis comprised the following: (1) 20 mg/day or 40 mg/day istradefylline use for PD; (2) placebo-controlled randomized trial with more than 10 subjects in each group; (3) assessment of off time or unified Parkinson’s disease rating scale (UPDRS) III during the on period; (4) written in English. A systematic literature search of PubMed, Web of Science and Cochrane Library was performed in May 2016 using the following syntax: (“Parkinson’s disease” or “PD”) and (“Istradefylline”) and (“randomized,” “random,” or “randomly”). As indicated in Fig. 1, six studies were finally included in the present meta-analysis. We contacted the corresponding author if incomplete data were detected. Three researchers independently performed the above-mentioned search and study selection. Finally, we resolved any discrepancies after discussion. Risk of bias was evaluated by the Cochrane Collaboration’s tool for risk of bias.

### Data Synthesis and Statistics

Detailed analysis methods are described in our previously published meta-analysis^20^. Briefly, we used the standardized mean difference (SMD) between the istradefylline and placebo groups, considering off time, UPDRS III score during the on phase, and UPDRS II score, to assess the effect of istradefylline 12 weeks after treatment. We estimated standard deviation (SD) for change from baseline based on a 95% confidence interval (CIs). In contrast to continuous data, a pooled risk ratio (RR) along with 95% CIs was calculated for dichotomous data. We investigated heterogeneity of the included studies with I-squared (I^2^). Adverse events described in more than 3 papers were defined as the targeted event. Sensitivity analyses were performed to establish robust evidence if there was a significant difference. All analyses were performed using Review Manager (RevMan 5.2) for Windows (http://ims.cochrane.org/revman) and R software (http://www.r-project.org/).

### Publication bias

In case of significant differences, publication bias was assessed by visual inspection and Egger’s test as described previously^22^.

## Results

### Study Characteristics and Risk of bias

Six placebo-controlled randomized studies met our inclusion criteria (n = 1175 istradefylline subjects, and n = 643 placebo subjects). The summary of the included studies is shown in supporting Table 1.

In terms of risk of bias, we cannot exclude the possibility of selection bias in several studies owing to a lack of description (please see supporting Fig. 1).

### Off time and UPDRS III

**Figure 2:**
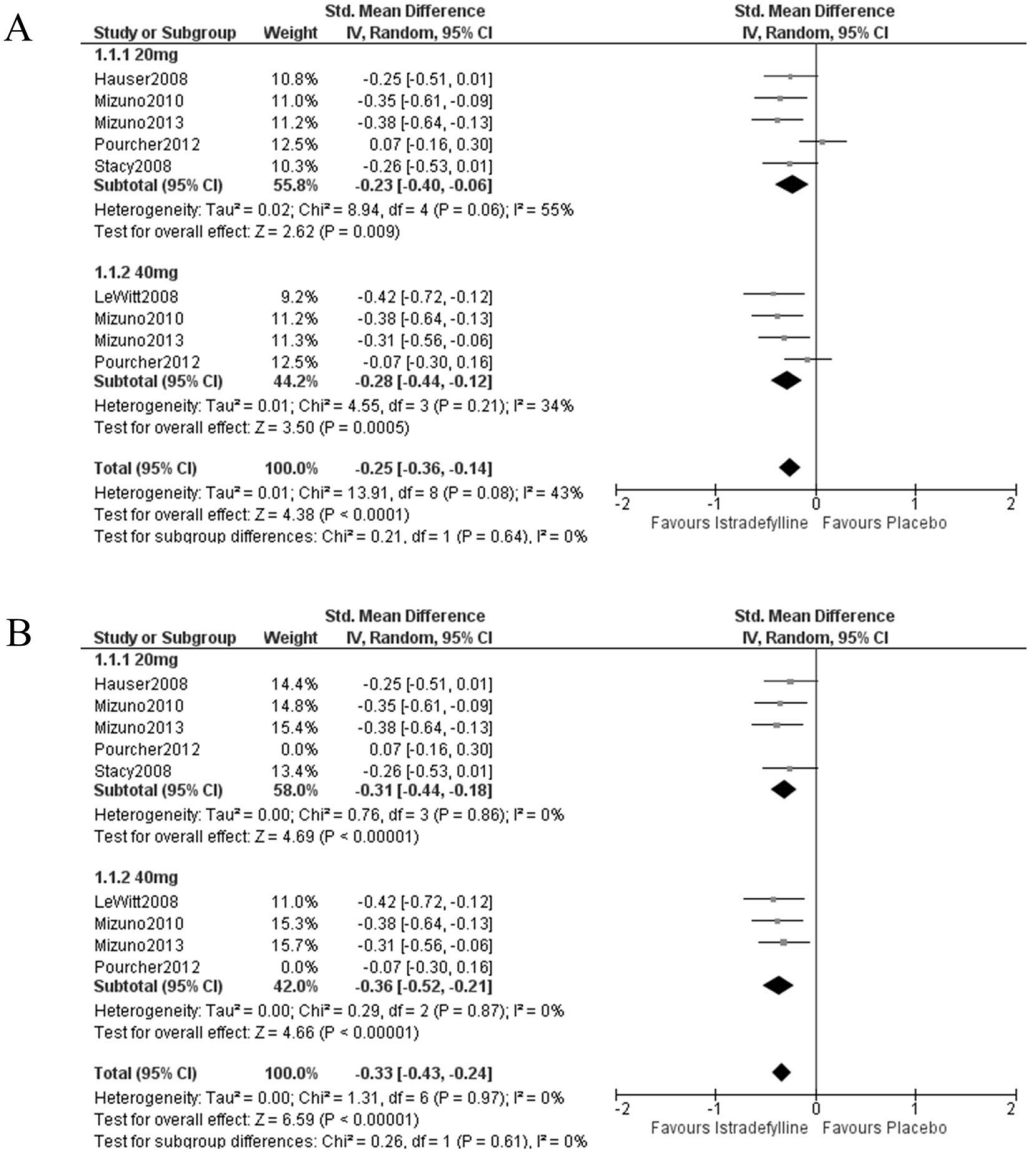
Forest plots of the standardized mean difference in off time. (**A**) Istradefylline 40 mg/day showed a significant reduction of off time in homogeneous studies. In contrast, istradefylline 20 mg/day showed a significant reduction of off time in heterogeneous studies. (**B**) Heterogeneity disappeared in istradefylline 20 mg/day use without “Pourcher 2012.”

For off time, the patients on 20 mg/day istradefylline showed significant improvement across heterogeneous studies (SMD = −0.23, 95% confidence interval (CI) = −0.40 to −0.06, *P* = 0.009, I^2^ = 55%, Fig. 2A). This heterogeneity disappeared when “Pourcher 2012” was excluded (I^2^ = 0%, Fig. 2B). In contrast, at 40 mg/day, istradefylline reduced off time across homogeneous studies (SMD = −0.28, 95% CI = −0.44 to −0.12, *P* = 0.0005, I^2^ = 34%, Fig. 2A). The significant effect of 20 mg/day istradefylline was lost without “Mizuno 2010” or “Mizuno 2013” (*P* = 0.06, supporting Table 2), but that of 40 mg/day was observed regardless of any exclusions (“LeWitt 2008,” *P* = 0.009; “Mizuno 2010,” *P* = 0.01; “Mizuno 2013,” *P* = 0.01; “Pourcher 2012,” *P* < 0.00001; supporting Table 2).

**Figure 3:**
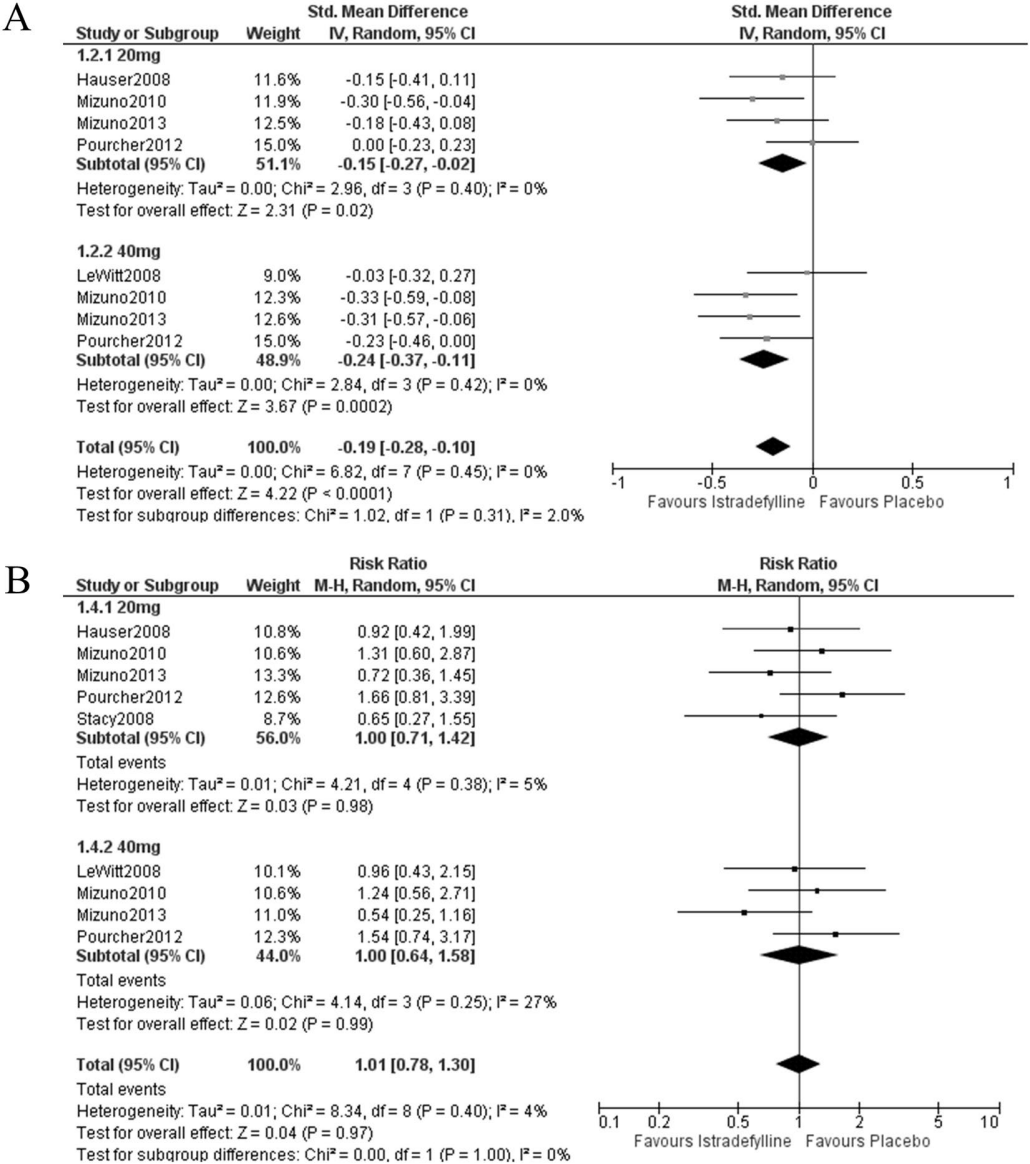
Forest plots of the standardized mean difference in unified Parkinson’s disease rating scale III (UPDRS III) and the pooled risk ratio of dropout. (**A**) Istradefylline improved UPDRS III regardless of any dosage in homogeneous studies. (**B**) There was no significant difference of the incidence of dropout between placebo and istradefylline. The included studies were homogeneous.

Istradefylline improved UPDRS III at on phase regardless of daily dose (20 mg, SMD = −0.15, 95% CI = −0.27 to −0.02, *P* = 0.02; 40 mg, SMD = −0.24, 95% CI = −0.37 to −0.11, *P* = 0.0002; Fig. 3A). These studies were homogeneous (I^2^ = 0%, Fig. 3A). Furthermore, sensitivity analysis revealed a robust effect of 40 mg/day istradefylline (“LeWitt 2008,” *P* < 0.0001; “Mizuno 2010,” *P* = 0.008; “Mizuno 2013,” *P* = 0.01; “Pourcher 2012,” *P* = 0.01; supporting Table 2). In contrast, there was no significant effect of istradefylline 20 mg/day without “Hauser 2008,” “Mizuno 2010,” or “Mizuno 2013” (“Hauser 2008,” *P* = 0.09; “Mizuno 2010,” *P* = 0.17; “Mizuno 2013,” *P* = 0.11; supporting Table 2).

No subgroup difference in off time or UPDRS III was seen between 20 mg/day and 40 mg/day istradefylline, which suggested that there was no dose effect (off time, I^2^ = 0%, Fig. 2A; UPDRS III, I^2^ = 2.0%; Fig. 3A).

### Tolerability

No significant difference was found between istradefylline and placebo groups in regard to dropouts (20 mg/day, *P* = 0.98; 40 mg/day, *P* = 0.99; Fig. 3B).

### UPDRS II

Istradefylline did not improve UPDRS II scores at any daily doses (20 mg, *P* = 0.64; 40 mg, *P* = 0.44; please see supporting Fig. 2).

### Adverse Events

**Figure 4:**
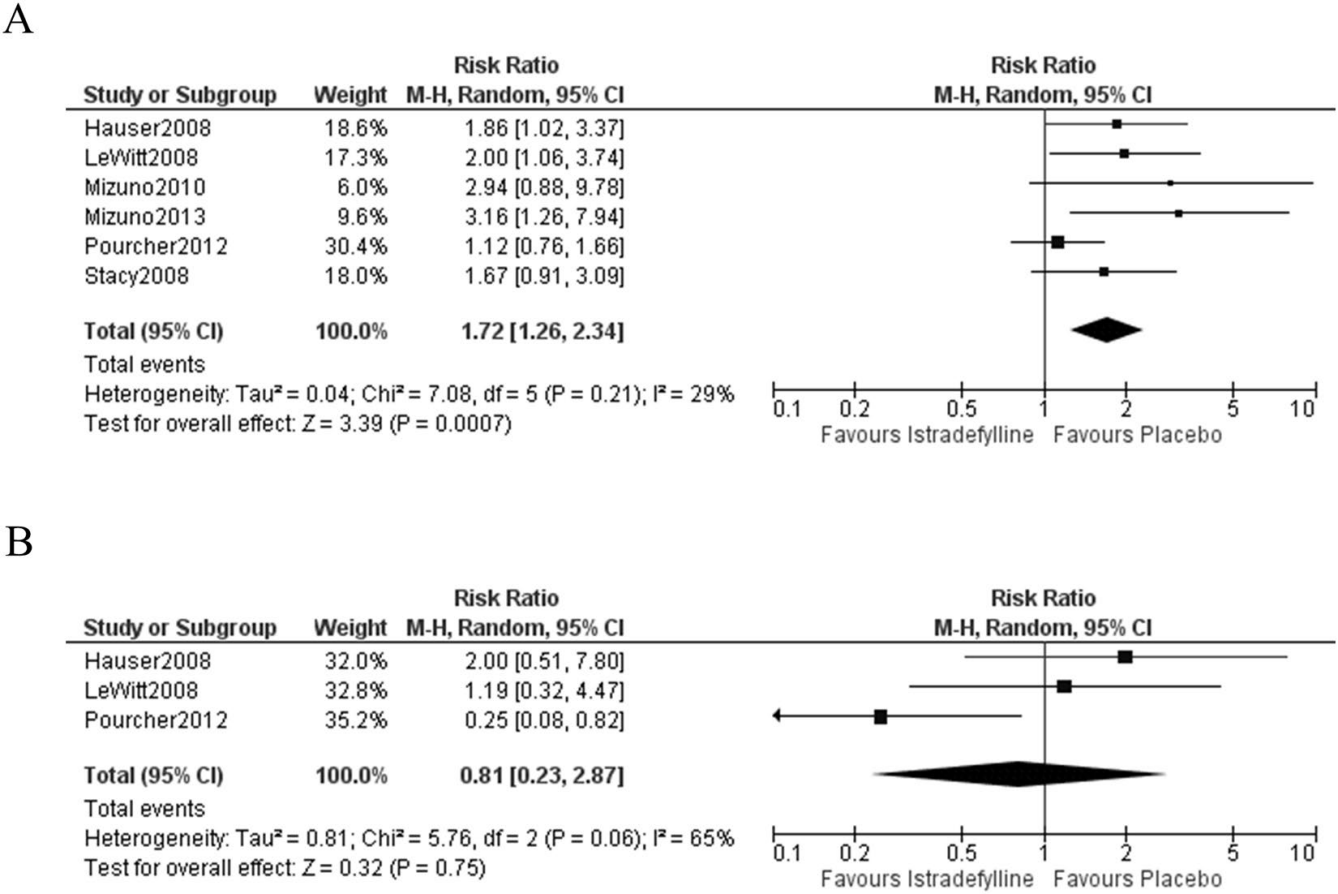
Forest plots of the pooled risk ratio of dyskinesia and tremor. (**A**) Homogeneous studies revealed that dyskinesia was worsened by istradefylline. (**B**) No significant difference was observed between groups for tremor.

Eight adverse events which satisfied our inclusion criteria were detected as targets. The aggravation of dyskinesia occurred more frequently in the istradefylline group than the placebo group (RR = 1.72, 95% CI = 1.26 to 2.34, *P* = 0.0007, Fig. 4A). The included studies were homogeneous (I^2^ = 29%, Fig. 4A). A significant effect of istradefylline was observed regardless of any exclusion (“Hauser 2008,” *P* = 0.005; “LeWitt 2008,” *P* < 0.006; “Mizuno 2010,” *P* = 0.002; “Mizuno 2013,” *P* = 0.002; “Pourcher 2012,” *P* < 0.0001; “Stacy 2008,” *P* = 0.004; supporting Table 2). The other motor symptom, tremor, showed no significant difference between istradefylline and placebo groups (RR = 0.81, 95% CI = 0.23 to 2.87, *P* = 0.75, Fig. 4B). There was no significant effect of istradefylline on the other adverse events (nausea, *P* = 0.41; constipation, *P* = 0.14; hallucination, *P* = 0.12; insomnia, *P* = 0.81; somnolence, *P* = 0.94; accident, *P* = 0.28, please see supporting Fig. 3).

### Publication bias

No clear asymmetry of funnel plots was identified, and there were no significant deviations of intercept in all outcomes except for dyskinesia (off time 20 mg/day, *P* = 0.12; off time 40 mg/day, *P* = 0.19; UPDRS III 20 mg/day, *P* = 0.16; UPDRS III 40 mg/day, *P* = 0.34). For dyskinesia, the funnel plot appeared asymmetrical (dyskinesia, *P* = 0.007, please see supporting Fig. 4).

## Discussion

Our meta-analysis revealed that 40 mg/day istradefylline could improve off time and motor symptoms, and both findings were supported by a sensitivity analysis. In terms of the effect of 20 mg/day istradefylline on off time, however, heterogeneity of the included studies was detected, but its cause remained unknown. Furthermore, we failed to provide robust evidence for 20 mg/day istradefylline for off time or UPDRS III using a sensitivity analysis. It was noted that “Pourcher 2012” contributed to significant heterogeneity for the effect of 20 mg/day istradefylline on off time. The characteristics of “Pourcher 2012” related to this included increased dosage of daily levodopa intake with a wider range, more men, and a longer duration compared to the other studies, which suggests that they analyzed effects in a more advanced stage of PD. These factors could thus affect the results. Taken together, istradefylline could be recommended for use in the early stages of PD. Given the results of the sensitivity analyses, it was possible that the effect of 20 mg/day istradefylline was smaller than that of 40 mg/day. In such a case, istradefylline at 20 mg/day produced variable results owing to the different background of each study, whereas istradefylline at 40 mg/day stably improved off time and motor symptoms. However, considering significant but weak effect on these symptoms, istradefylline appeared suitable for adjuvant therapy at advanced stage in clinical practice.

Istradefylline was also administered at the advanced stage when dyskinesia was seen as motor complication owing to levodopa treatment. PD patients with levodopa-induced dyskinesia were reported to show elevated A_2A_ receptor binding^23^. Given this finding, A_2A_ receptor antagonists would be expected not to worsen dyskinesia. However, the exaggeration of dyskinesia was demonstrated by our meta-analysis; at the same time, publication bias could have contributed to this effect. Additional studies need to be performed to confirm the effect of istradefylline on levodopa-induced dyskinesia. There was no difference of the proportion of dropouts between istradefylline and placebo groups, which might indicate adequate tolerability in spite of the aggravation of dyskinesia.

No non-motor symptoms were worsened by istradefylline as in supporting Fig. 3. Administration of istradefylline was reported to improve overactive bladder and daytime sleepiness^24,25^, however, these were not randomized placebo-controlled trials. Further studies are needed to establish robust evidence of efficacy of istradefylline on non-motor symptoms.

A previous meta-analysis reported that coffee could reduce the risk of PD^9^. Caffeine is considered a nonspecific A_2A_ receptor antagonist^8^, and istradefylline is expected to have a similar effect to caffeine on neurons, i.e. a neuroprotective effect. Indeed, several papers demonstrated that A_2A_ receptor antagonists prevented neurodegeneration in dopaminergic neurons in MPTP and 6-OHDA rodent models of PD^26,27^. Deletion of the A_2A_ receptor inhibited loss of dopamine in the striatum, and of dopaminergic neurons in the SNc^8^. Furthermore, there was a tendency for caffeine to reduce the risk of developing dyskinesia in an observational study^10^. In this sense, istradefylline could be recommended for use before onset of dyskinesia or wearing off, to promote disease modification owing to neuroprotection of dopaminergic neurons; this is the case despite all previous randomized controlled trials (RCT) targeting patients with PD in an advanced phase.

Although istradefylline was known to block A_2A_ receptors in the indirect pathway, the network-level mode of action for istradefylline has remained an enigma. Prior reports identified abnormal brain networks in PD^28,29^, for which the elucidation of the effect of istradefylline could lead to the development of novel adenosinergic therapies.

The previous meta-analysis reported significant effect of 40 mg/day istradefylline on reduction of off time with heterogeneous studies, but failed to demonstrate that 20 mg/day istradefylline could improve motor symptoms^18^. Additional studies and usage of standardized value allowed us to reveal significant effects of istradefylline with homogeneous studies. The assessment of a funnel plot revealed that summary effect of istradefylline on dyskinesia could be biased by publication. Moreover, the present research added new evidence of favorable tolerability.

This meta-analysis had multiple limitations. First, it was possible that limiting the search to studies written in English, and the modest number of included studies, might contribute to bias. Second, Egger’s test detected publication bias in the analysis of dyskinesia. Third, the included studies examined the short-term effect of istradefylline on patients with PD. One open-label study showed the long-term effect of istradefylline^30^, but we require an RCT with long-term follow up to reveal the longitudinal outcome including the putative neuroprotective effect.

In summary, the present meta-analysis has provided robust evidence that istradefylline at 20 mg/day improves UPDRS III, and at 40 mg/day improves both off time and UPDRS III. The latter findings were further supported by sensitivity analyses. There was no significant difference in dropout ratio between istradefylline and placebo groups, which suggested this drug was well tolerated. However, dyskinesia could be worsened, but publication bias was detected, leaving the issue murky. Istradefylline at 40 mg/day is the only drug to have meta-analysis-based evidence among non-dopaminergic drugs potentially useful in PD, and is recommended for administration in the early phases of PD, or at least before the onset of motor complications.

## Electronic supplementary material

Supplementary information
