# Supplementary material for: The effect of istradefylline for Parkinson’s disease: A meta-analysis
Source: Sci Rep. 2017 Dec 21;7:18018. doi: 10.1038/s41598-017-18339-1 (PMC5740127; doi:10.1038/s41598-017-18339-1)
Supplement: Supplementary file 1 — Supplementary information [file 41598_2017_18339_MOESM1_ESM.pdf]

The effect of istradefylline for Parkinson's disease: A meta-analysis

Wataru Sako,[1*] Nagahisa Murakami,[1] Keisuke Motohama,[1,2] Yuishin Izumi,[1] Ryuji Kaji[1]

[1] Department of Clinical Neuroscience, Institute of Biomedical Sciences, Tokushima University Graduate School, Tokushima, Japan

[2] Student Laboratory, Faculty of Medicine, Tokushima University, Tokushima, Japan

Address correspondence to:
Wataru Sako, MD, PhD
2-50-1 Kuramoto-cho, Tokushima, Japan 770-8503
Phone: 81-88-633-7207; Fax: 81-88-633-7208; Email: dwsako@tokushima-u.ac.jp

SUPPORTING TABLES

Supporting Table 1. Characteristics of studies included in this meta-analysis.

Study Name	Group (Istradefylline dose mg/day)	Sample Size	Drop out	Age - yr	Male (Female)	Time since diagnosis - years	Time since onset of motor complication - years	Daily levodopa intake -mg/day	off-time -hours	on-period UPDRS III	Follow-up time points - week
**Hauser 2008¶**	Istradefylline (20)	115	11	63 ± 9.5	76 (39)	10.0 ± 5.5	4.0 ± 3.7	652 ± 370.6	6.7 ± 2.8	23.9 ± 11.3	12
	Placebo	115	12	64 ± 10.2	77 (38)	8.8 ± 4.4	3.6 ± 3.3	631 ± 356.7	6.5 ± 2.1	22.8 ± 11.2	12
**LeWitt 2008§**	Istradefylline (40)	129	15	63 ± 9	77 (52)	9.3 ± 4.7	3.3 ± 2.5	560 ± 291	6.4 ± 2.7	17.7 ± 11.1	12
	Placebo	66	8	64 ± 10	40 (26)	9.3 ± 5.1	3.6 ± 3.2	589 ± 301	6.2 ± 2.5	18.0 ± 11.2	12
**Stacy 2008§**	Istradefylline (20)	163	11	65.0 ± 9.59	104 (59)	3.71 ± 3.830	NA	NA	5.72 ± 2.502	17.6 ± 9.82	12
	Placebo	77	8	63.0 ± 12.05	54 (23)	3.81 ± 2.694	NA	NA	6.31 ± 2.591	16.2 ± 8.80	12
**Mizuno 2010†**	Istradefylline (40)	124	13	63.7 ± 8.6	55 (69)	8.089 ± 4.048	3.126 ± 2.884	415.3 ± 159.2	6.55 ± 2.48	21.1 ± 11.0	12
	Istradefylline (20)	115	13	65.1 ± 7.2	50 (65)	8.037 ± 4.076	3.167 ± 2.499	407.0 ± 113.1	6.79 ± 2.86	21.0 ± 10.6	12
	Placebo	118	10	65.0 ± 7.6	45 (73)	8.338 ± 4.826	3.506 ± 3.015	426.3 ± 143.0	6.43 ± 2.71	20.6 ± 9.2	12
**Pourcher 2012¶**	Istradefylline (40)	152	17	63 ± 9.3	100 (52)	8.5 ± 4.6	3.4 ± 3.2	645 ± 407	6.9 ± 2.3	21.9 ± 11.5	12
	Istradefylline (20)	149	18	64 ± 9.8	103 (46)	8.9 ± 4.6	3.7 ± 3.4	602 ± 357	6.7 ± 2.2	22.3 ± 11.3	12
	Placebo	151	14	63 ± 8.3	97 (54)	9.1 ± 5.1	3.7 ± 3.5	718 ± 394	6.7 ± 2.1	22.7 ± 11.8	12
**Mizuno 2013†**	Istradefylline (40)	123	9	65.7 ± 9.0	64 (59)	7.730 ± 4.547	3.258 ± 3.009	420.5 ± 131.8	5.97 ± 2.45	20.7 ± 11.0	12
	Istradefylline (20)	120	12	66.1 ± 8.6	40 (80)	7.301 ± 4.206	3.187 ± 2.759	430.8 ± 156.5	6.55 ± 2.72	21.3 ± 10.8	12

	Placebo	123	17	65.8 ± 8.6	58 (65)	7.990 ± 4.453	3.432 ± 3.470	425.4 ± 146.4	6.31 ± 2.47	21.6 ± 11.6	12

Abbreviations: UPDRS, unified Parkinson's disease rating scale; NA, not available. ¶: safety analysis set; §: intent to treat; †: full analysis set.

Supporting Table 2. Results of sensitivity analysis.

Outcome	Excluded Study	Sample size (Istradefylline, Placebo)	SMD or RR [95% CI]	Test for Heterogeneity		Test for Overall Effect	
				I^2 - %	***P***	Z	***P***
**Off time 20mg**	**Hauser 2008**	542, 464	-0.22 [-0.44, -0.01]	66	0.03	2.02	0.04
	**Mizuno 2010**	539, 459	-0.20 [-0.40, 0.01]	61	0.05	1.91	0.06
	**Mizuno 2013**	534, 454	-0.19 [-0.38, 0.01]	57	0.07	1.91	0.06
	**Pourcher 2012**	510, 431	-0.31 [-0.44, -0.18]	0	0.86	4.59	<0.00001
	**Stacy 2008**	491, 500	-0.22 [-0.44, -0.01]	66	0.03	2.02	0.04
**Off time 40mg**	**LeWitt 2008**	392, 387	-0.25 [-0.44, -0.06]	43	0.17	2.61	0.009
	**Mizuno 2010**	397, 335	-0.25 [-0.45, -0.05]	45	0.16	2.45	0.01
	**Mizuno 2013**	398, 330	-0.28 [-0.50, -0.05]	55	0.11	2.44	0.01
	**Pourcher 2012**	376, 307	-0.36 [-0.52, -0.21]	0	0.87	4.66	<0.00001
**UPDRS III 20mg**	**Hauser 2008**	379, 387	-0.15 [-0.32, 0.02]	32	0.23	1.70	0.09
	**Mizuno 2010**	376, 382	-0.10 [-0.24, 0.04]	0	0.55	1.38	0.17
	**Mizuno 2013**	371, 377	-0.14 [-0.31, 0.03]	31	0.24	1.60	0.11
	**Pourcher 2012**	347, 354	-0.21 [-0.36, -0.06]	0	0.69	2.75	0.006
**UPDRS III 40mg**	**LeWitt 2008**	392, 387	-0.29 [-0.43, -0.15]	0	0.81	3.98	<0.0001
	**Mizuno 2010**	397, 335	-0.21 [-0.36, -0.05]	6	0.34	2.65	0.008
	**Mizuno 2013**	398, 330	-0.21 [-0.37, -0.05]	16	0.30	2.55	0.01
	**Pourcher 2012**	376, 307	-0.24 [-0.42, -0.06]	29	0.24	2.57	0.01
**Dyskinesia**	**Hauser 2008**	1083, 539	1.74 [1.18, 2.57]	41	0.14	2.78	0.005

**LeWitt 2008**	1069, 588	1.70 [1.17, 2.48]	39	0.16	2.77	0.006
**Mizuno 2010**	955, 535	1.66 [1.20, 2.29]	33	0.20	3.06	0.002
**Mizuno 2013**	951, 528	1.56 [1.17, 2.09]	15	0.32	3.04	0.002
**Pourcher 2012**	897, 503	2.02 [1.47, 2.78]	0	0.78	4.35	<0.0001
**Stacy 2008**	1035, 577	1.79 [1.20, 2.66]	44	0.13	2.88	0.004

Abbreviations: RR, risk ratio; CI, confidence interval; SMD, standardized mean difference; UPDRS, unified Parkinson's disease rating scale.

SUPPORTING FIGURE LEGENDS

Supporting Figure 1. (A) Risk of bias assessment for the included studies. Each risk of bias item was presented as percentages across all included studies. (B) Risk of bias summary. Each risk of bias item for each included study is included.

Supporting Figure 2. Forest plot of the standardized mean difference in unified Parkinson's disease rating scale II (UPDRS II). There was no significant difference in UPDRS II between placebo and istradefylline.

Supporting Figure 3. Forest plots of the pooled risk ratio of adverse events. No significant difference was observed in nausea (A), constipation (B), hallucination (C), insomnia (D), somnolence (E), and accident (F) between placebo and istradefylline.

Supporting Figure 4. Funnel plot of comparison for dyskinesia. The funnel plot appeared asymmetric and a significant deviation of intercept from zero was found.

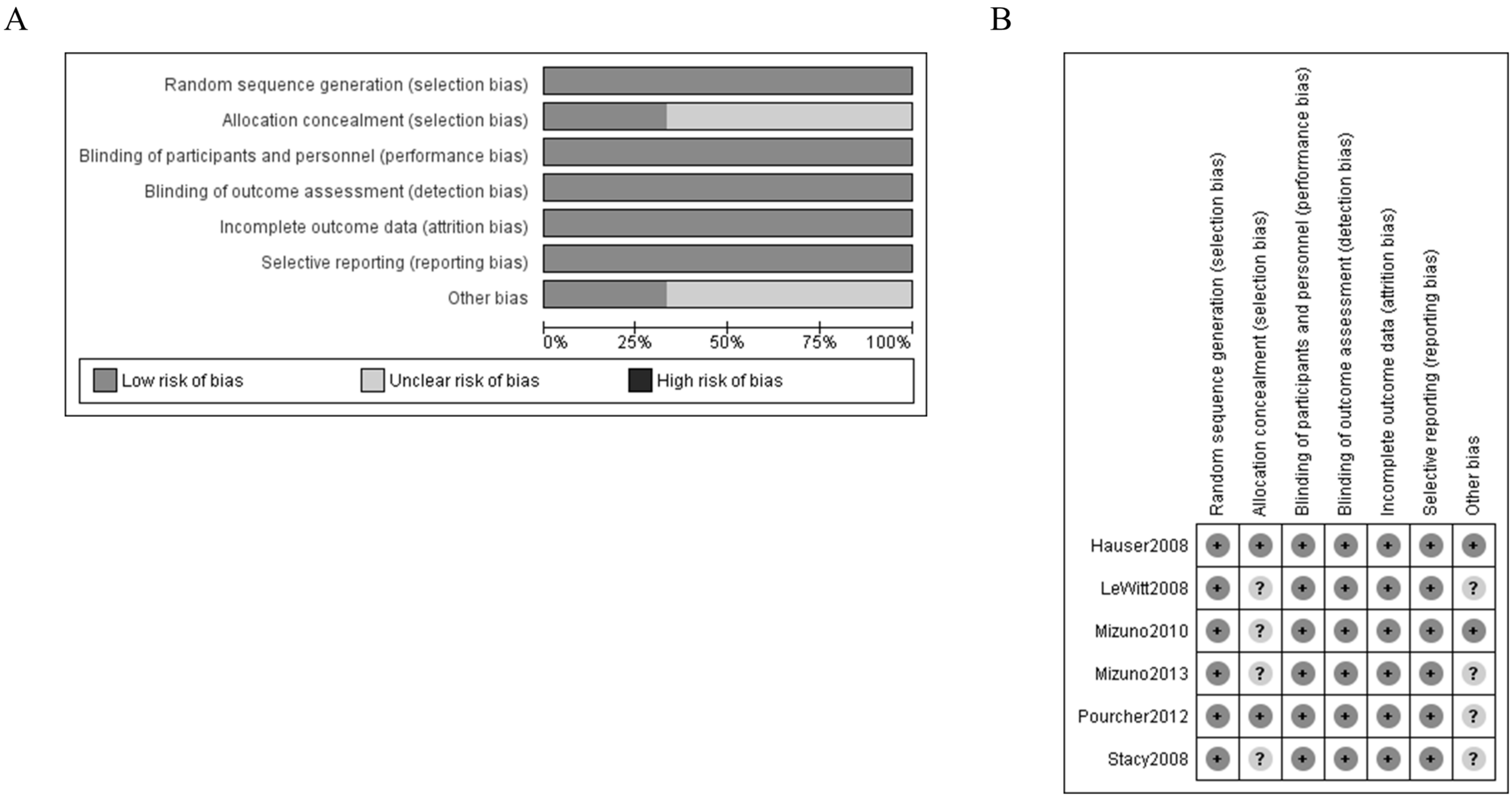
A
Random sequence generation (selection bias)
Allocation concealment (selection bias)
Blinding of participants and personnel (performance bias)
Blinding of outcome assessment (detection bias)
Incomplete outcome data (attrition bias)
Selective reporting (reporting bias)
Other bias
0%
25%
50%
75%
100%
Low risk of bias
Unclear risk of bias
High risk of bias
B
Random sequence generation (selection bias)
Allocation concealment (selection bias)
Blinding of participants and personnel (performance bias)
Blinding of outcome assessment (detection bias)
Incomplete outcome data (attrition bias)
Selective reporting (reporting bias)
Other bias
Hauser2008
LeWitt2008
Mizuno2010
Mizuno2013
Pourcher2012
Stacy2008

Study or Subgroup	Weight	Std. Mean Difference IV, Random, 95% CI
**1.3.1 20mg**		
Hauser2008	22.9%	0.18 [-0.08, 0.44]
Pourcher2012	29.6%	-0.05 [-0.28, 0.18]
**Subtotal (95% CI)**	**52.5%**	**0.05 [-0.17, 0.28]**
Heterogeneity: Tau² = 0.01; Chi² = 1.69, df = 1 (P = 0.19); I² = 41%		
Test for overall effect: Z = 0.47 (P = 0.64)		
**1.3.2 40mg**		
LeWitt2008	17.8%	-0.14 [-0.44, 0.15]
Pourcher2012	29.7%	-0.03 [-0.26, 0.20]
**Subtotal (95% CI)**	**47.5%**	**-0.07 [-0.25, 0.11]**
Heterogeneity: Tau² = 0.00; Chi² = 0.38, df = 1 (P = 0.54); I² = 0%		
Test for overall effect: Z = 0.77 (P = 0.44)		
**Total (95% CI)**	**100.0%**	**-0.01 [-0.13, 0.12]**
Heterogeneity: Tau² = 0.00; Chi² = 2.93, df = 3 (P = 0.40); I² = 0%		
Test for overall effect: Z = 0.14 (P = 0.89)		
Test for subgroup differences: Chi² = 0.71, df = 1 (P = 0.40), I² = 0%		

Std. Mean Difference
IV, Random, 95% CI

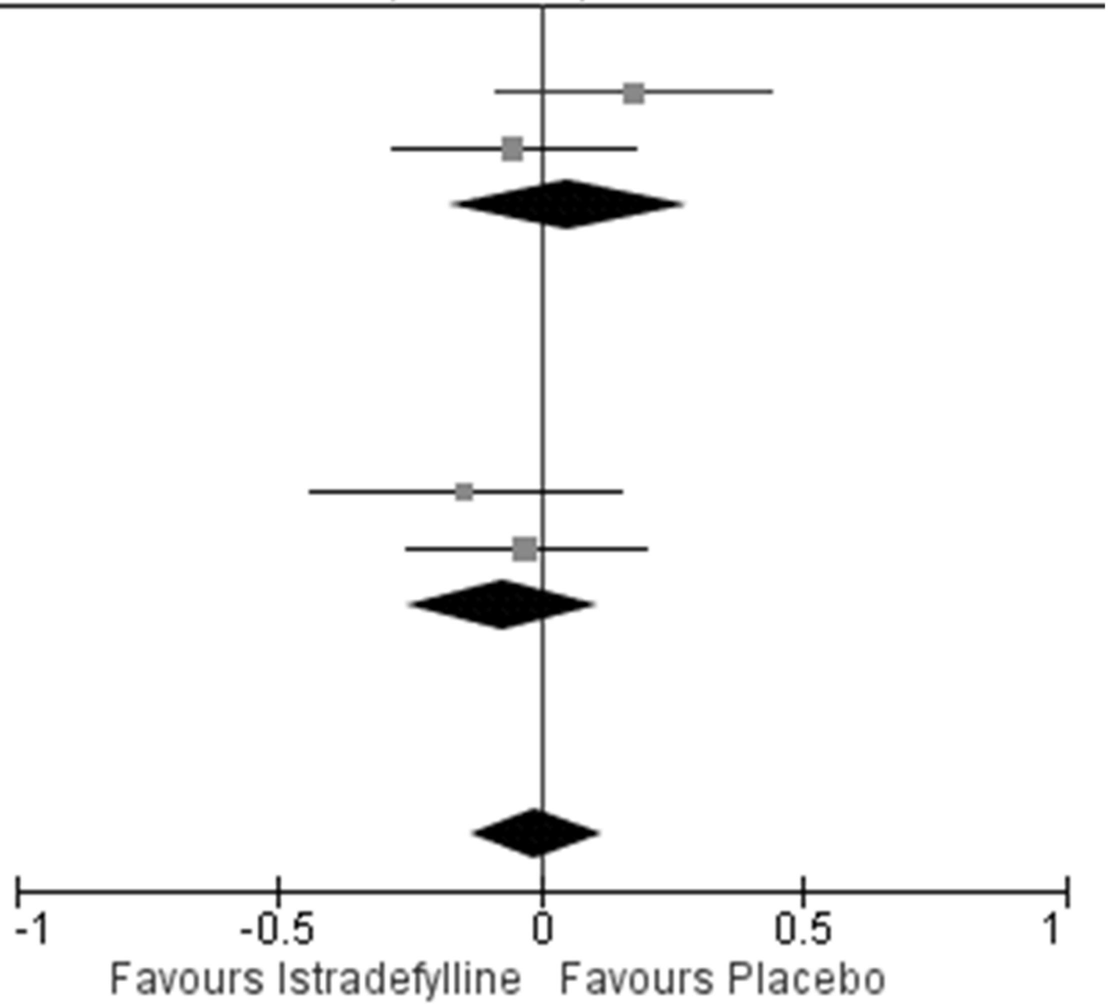

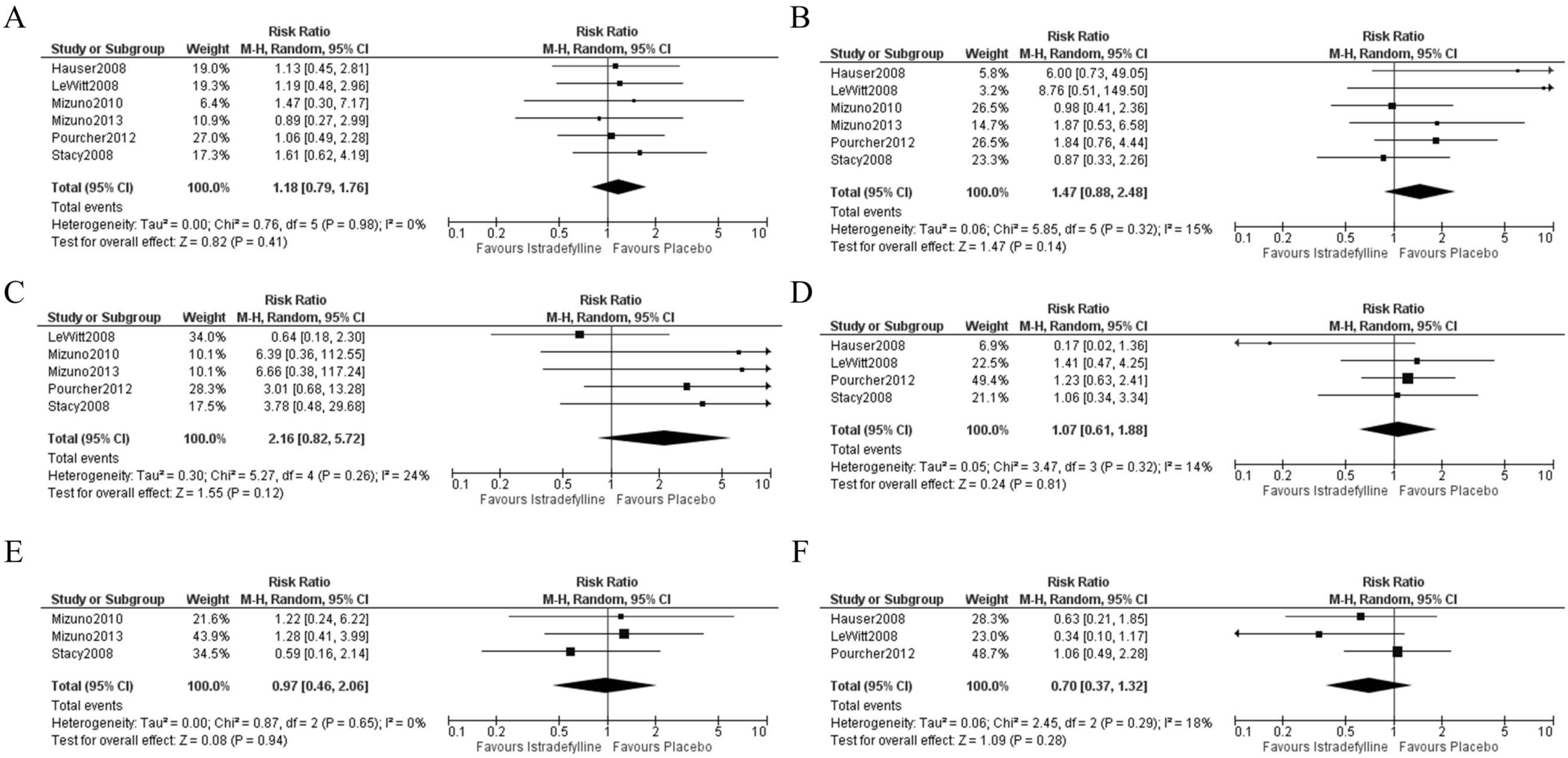
A
Risk Ratio
Risk Ratio
Study or Subgroup
Weight
M-H, Random, 95% CI
M-H, Random, 95% CI
Hauser2008 19.0% 1.13 [0.45, 2.81]
LeWitt2008 19.3% 1.19 [0.48, 2.96]
Mizuno2010 6.4% 1.47 [0.30, 7.17]
Mizuno2013 10.9% 0.89 [0.27, 2.99]
Pourcher2012 27.0% 1.06 [0.49, 2.28]
Stacy2008 17.3% 1.61 [0.62, 4.19]
Total (95% CI) 100.0% 1.18 [0.79, 1.76]
Total events
Heterogeneity: Tau² = 0.00; Chi² = 0.76, df = 5 (P = 0.98); I² = 0%
Test for overall effect: Z = 0.82 (P = 0.41)
0.1 0.2 0.5 1 2 5 10
Favours Istradefylline Favours Placebo
B
Risk Ratio
Risk Ratio
Study or Subgroup
Weight
M-H, Random, 95% CI
M-H, Random, 95% CI
Hauser2008 5.8% 6.00 [0.73, 49.05]
LeWitt2008 3.2% 8.76 [0.51, 149.50]
Mizuno2010 26.5% 0.98 [0.41, 2.36]
Mizuno2013 14.7% 1.87 [0.53, 6.58]
Pourcher2012 26.5% 1.84 [0.76, 4.44]
Stacy2008 23.3% 0.87 [0.33, 2.26]
Total (95% CI) 100.0% 1.47 [0.88, 2.48]
Total events
Heterogeneity: Tau² = 0.06; Chi² = 5.85, df = 5 (P = 0.32); I² = 15%
Test for overall effect: Z = 1.47 (P = 0.14)
0.1 0.2 0.5 1 2 5 10
Favours Istradefylline Favours Placebo
C
Risk Ratio
Risk Ratio
Study or Subgroup
Weight
M-H, Random, 95% CI
M-H, Random, 95% CI
LeWitt2008 34.0% 0.64 [0.18, 2.30]
Mizuno2010 10.1% 6.39 [0.36, 112.55]
Mizuno2013 10.1% 6.66 [0.38, 117.24]
Pourcher2012 28.3% 3.01 [0.68, 13.28]
Stacy2008 17.5% 3.78 [0.48, 29.68]
Total (95% CI) 100.0% 2.16 [0.82, 5.72]
Total events
Heterogeneity: Tau² = 0.30; Chi² = 5.27, df = 4 (P = 0.26); I² = 24%
Test for overall effect: Z = 1.55 (P = 0.12)
0.1 0.2 0.5 1 2 5 10
Favours Istradefylline Favours Placebo
D
Risk Ratio
Risk Ratio
Study or Subgroup
Weight
M-H, Random, 95% CI
M-H, Random, 95% CI
Hauser2008 6.9% 0.17 [0.02, 1.36]
LeWitt2008 22.5% 1.41 [0.47, 4.25]
Pourcher2012 49.4% 1.23 [0.63, 2.41]
Stacy2008 21.1% 1.06 [0.34, 3.34]
Total (95% CI) 100.0% 1.07 [0.61, 1.88]
Total events
Heterogeneity: Tau² = 0.05; Chi² = 3.47, df = 3 (P = 0.32); I² = 14%
Test for overall effect: Z = 0.24 (P = 0.81)
0.1 0.2 0.5 1 2 5 10
Favours Istradefylline Favours Placebo
E
Risk Ratio
Risk Ratio
Study or Subgroup
Weight
M-H, Random, 95% CI
M-H, Random, 95% CI
Mizuno2010 21.6% 1.22 [0.24, 6.22]
Mizuno2013 43.9% 1.28 [0.41, 3.99]
Stacy2008 34.5% 0.59 [0.16, 2.14]
Total (95% CI) 100.0% 0.97 [0.46, 2.06]
Total events
Heterogeneity: Tau² = 0.00; Chi² = 0.87, df = 2 (P = 0.65); I² = 0%
Test for overall effect: Z = 0.08 (P = 0.94)
0.1 0.2 0.5 1 2 5 10
Favours Istradefylline Favours Placebo
F
Risk Ratio
Risk Ratio
Study or Subgroup
Weight
M-H, Random, 95% CI
M-H, Random, 95% CI
Hauser2008 28.3% 0.63 [0.21, 1.85]
LeWitt2008 23.0% 0.34 [0.10, 1.17]
Pourcher2012 48.7% 1.06 [0.49, 2.28]
Total (95% CI) 100.0% 0.70 [0.37, 1.32]
Total events
Heterogeneity: Tau² = 0.06; Chi² = 2.45, df = 2 (P = 0.29); I² = 18%
Test for overall effect: Z = 1.09 (P = 0.28)
0.1 0.2 0.5 1 2 5 10
Favours Istradefylline Favours Placebo

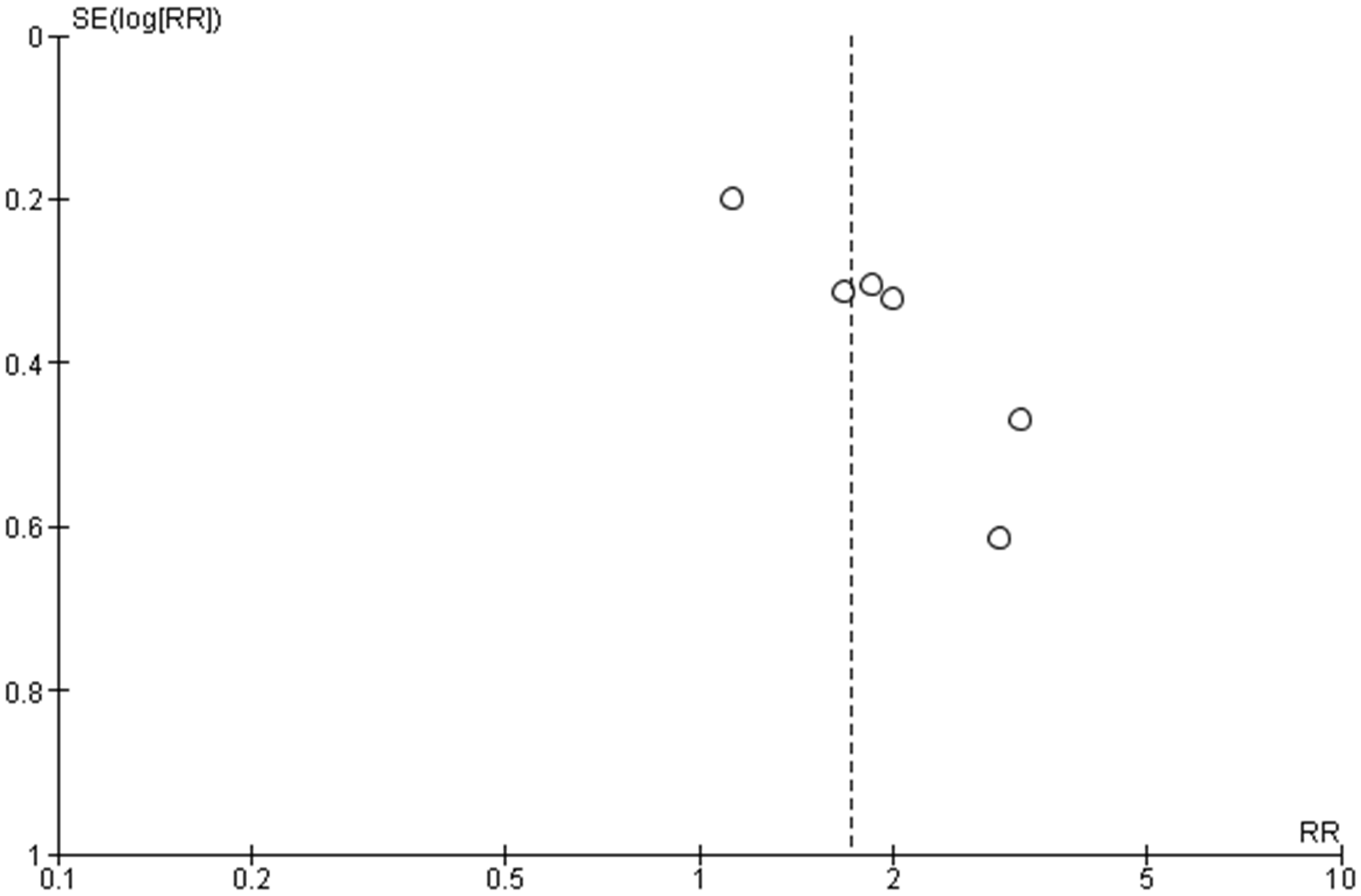
SE(log[RR])
0
0.2
0.4
0.6
0.8
1
RR
0.1
0.2
0.5
1
2
5
10
